# Recent Aspects on the Pathogenesis Mechanism, Animal Models and Novel Therapeutic Interventions for Middle East Respiratory Syndrome Coronavirus Infections

**DOI:** 10.3389/fmicb.2019.00569

**Published:** 2019-03-26

**Authors:** Sinosh Skariyachan, Sneha Basavaraj Challapilli, Swathi Packirisamy, Supreetha Toplar Kumargowda, Vaishnavi Sneha Sridhar

**Affiliations:** R&D Centre, Department of Biotechnology, Dayananda Sagar College of Engineering, Bengaluru, India

**Keywords:** MERS-CoV, emerging zoonotic virus, mechanisms of pathogenesis, animal models, probable drug targets, vaccine development

## Abstract

Middle East Respiratory Syndrome Coronavirus (MERS-CoV) is an emerging zoonotic virus considered as one of the major public threat with a total number of 2 298 laboratory-confirmed cases and 811 associated deaths reported by World Health Organization as of January 2019. The transmission of the virus was expected to be from the camels found in Middle Eastern countries via the animal and human interaction. The genome structure provided information about the pathogenicity and associated virulent factors present in the virus. Recent studies suggested that there were limited insight available on the development of novel therapeutic strategies to induce immunity against the virus. The severities of MERS-CoV infection highlight the necessity of effective approaches for the development of various therapeutic remedies. Thus, the present review comprehensively and critically illustrates the recent aspects on the epidemiology of the virus, the structural and functional features of the viral genome, viral entry and transmission, major mechanisms of pathogenesis and associated virulent factors, current animal models, detection methods and novel strategies for the development of vaccines against MERS-CoV. The review further illustrates the molecular and computational virtual screening platforms which provide insights for the identification of putative drug targets and novel lead molecules toward the development of therapeutic remedies.

## Introduction

The coronaviruses such as HCoV-229E, HCoV-NL63 (α-coronavirus) and HCoV-OC43 and HKU1 (β-coronavirus) circulated in the human population and caused mild respiratory infections (Hamre and Procknow, 1966; Fouchier et al., 2004; van der Hoek et al., 2004; Woo et al., 2005). Over the last two decades, there have been two zoonotic emergence of coronaviruses into the human population, both linked with Severe Acute Respiratory Syndrome Coronavirus (SARS-CoV) (Drosten et al., 2003; Kuiken et al., 2003) and Middle East Respiratory Syndrome Coronavirus (MERS-CoV) (Zaki et al., 2012; Chafekar and Fielding, 2018).

Middle East Respiratory Syndrome Coronavirus belonged to β-coronavirus, clade-c (van Boheemen et al., 2012; de Groot et al., 2013) and was initially known as Human Coronavirus Erasmus Medical Center/2012. or HCoV-EMC/2012 (Chan et al., 2012). MERS-CoV infections resulted high mortality rate in human, and till date, very limited therapeutic intervention or vaccine are available for the treatment of infections caused by the virus (Mustafa et al., 2017; Hui et al., 2018).

Middle East Respiratory Syndrome Coronavirus found in various natural hosts such as the dromedary calves (*Camelus dromedarius*), bats (*Vespertilio superans* and *Neoromicia capensis*), and European hedgehog (*Erinaceus europaeus*) (Zhang et al., 2016; Lau et al., 2017). The replication of the virus has observed in various other animals such as rabbit, goat, civet, pig, camelid, and horse (Muller et al., 2012; Chan et al., 2013a; Al-Tawfiq et al., 2014; Eckerle et al., 2014; Meyer et al., 2015). The prevalence MERS-CoV was recently reported in non-camelid domestic mammals such as sheep, goat, donkey, and cow that were in contact with camels (Kandeil et al., 2019). The origin of human MERS-CoV is considered to be from the bats. Several coronaviruses were isolated and found that which were genetically similar to human MERS-CoV. Phylogenetic analysis of MERS-CoV showed that a close relationship to Pipistrellus bat coronavirus HKU5 and Tylonycteris bat coronavirus HKU4 (Selinger et al., 2014). Studies have suggested that there were few evidences where the virus showed camel origin (Zhang et al., 2016). Studies revealed that the sero-prevalence of the virus was greater among the people those who were exhibited direct and indirect contact with dromedary camels when compared to those who were exhibited with the general population (Watson et al., 2014; Zhou et al., 2015; Conzade et al., 2018). Recent study revealed that the prevalence of MERS-CoV infection in camel workers was found to be very high in Saudi Arabia (Alshukairi et al., 2018). Thus, this review provide the recent perspectives on the epidemiology and genome composition of MERS-CoV with a special emphasis on the viral entry, clinical manifestations, mechanism of pathogenesis, animal models, detection, and development of vaccines with an insight on various computational biology perspectives.

## Epidemiology

The first case of MERS-CoV infection was reported in Saudi Arabia in 2012 (Zaki et al., 2012). From the period of its emergence, the virus has infected and propagated across more than 20 countries, contributing to a wide number of laboratory-confirmed cases. In 2015, the MERS-CoV outbreak in South Korea involved 186 cases which included 38 fatalities. A total of 83% cases of transmission were due to five super spreaders, and 44% of 186 cases were in the patients those who were experienced nosocomial transmission at 16 hospitals. The epidemic lasted for 2 months and the government authorities quarantined 16,993 persons for 14 days to manage the outbreak. This outbreak provides an exclusive opportunity to seal the gap in our awareness of MERS-CoV infection (Oh et al., 2018). Recent statistics suggested that as of 31 December 2018, the total of 2,279 laboratory-confirmed cases of MERS-CoV with 806 associated deaths (case fatality rate: 35.4%) were reported in 27 countries [Harcourt et al., 2018; World Health Organization (WHO), 2018a]. Among various cases, 1901 confirmed cases with 732 related deaths (fatality rate 38.5%) were reported from Saudi Arabia [Harcourt et al., 2018; World Health Organization (WHO), 2018a].

In a comprehensive report by WHO, 1841 laboratory cases were confirmed between 2012 and 2016 in which 80% of them were reported from Kingdom of Saudi Arabia. The remaining cases were reported from 27 countries in Middle East, Asia, North Africa, United States of America, and Europe [World Health Organization (WHO), 2016a]. A total of 13 cases were reported between October to November 2017, amongst which 12 cases were reported in male and only one case was reported in female. Most of the infected individuals were hospitalized due to the contact with camels and consumption of camel milk with the virus [World Health Organization (WHO), 2017a]. Additionally, two laboratory cases were also reported in which the patients belonged to the countries such as Malaysia and United Arab Emirates [World Health Organization (WHO), 2018a]. The laboratory confirmed cases and deaths reported in several countries from the year 2012 to 2018 are shown in Table 1.

**Table 1:** Laboratory confirmed cases of MERS-CoV infections and deaths reported in several countries from the year 2012 to 2018.

Year	Region	MERS-CoV infections	Deaths due to MERS-CoV infections	Reference
2012	Saudi Arabia, Qatar, and Jordan	9	3	World Health Organization, 2012
2013	Saudi Arabia, United Kingdom, France, Tunisia, and Italy	167	71	World Health Organization (WHO), 2013a
2014	Saudi Arabia, United Arab Emirates, Jordan, Iran, and Egypt	765	273	World Health Organization, 2014
2015	Saudi Arabia, Republic of Korea, Jordan, Kuwait, The Philippines, Thailand, United Arab Emirates, Oman, Qatar, China, and Iran	680	237	World Health Organization, 2015a,b
2016	Saudi Arabia, United Arab Emirates, Qatar, Lebanon, and Oman	243	75	World Health Organization (WHO), 2016b
2017	Saudi Arabia, United Arab Emirates, Qatar, Lebanon, and Oman	258	81	World Health Organization (WHO), 2017b
2018	Saudi Arabia and Malaysia, United Arab Emirates	96	41	World Health Organization (WHO), 2018a


## The Genome of MERS-CoV

In the early years of 2000, coronaviruses were not acquired great importance in research and development. With the emergence of SARS in the year 2002 and MERS in 2012, the interest of many researchers was stimulated toward coronavirus (Perlman, 2015).

The morphology of coronavirus includes spherical or pleomorphic shapes with a diameter of 80–120 nm (Masters, 2006). The genome of the coronavirus consists of 6 and 7 open reading frames (ORFs). The ORF 1a and 1b encompass two-third of the viral genome which encodes the non-structural poly-proteins and the other four ORFs on the downstream side encode for the structural proteins such as envelope protein (E), Spike protein (S), nucleocapsid protein (N), and membrane protein (M). In some coronaviruses, hemagglutinin-esterase (HE) gene is present in the region between ORF 1b and S (Marra et al., 2003; Rota et al., 2003; Snijder et al., 2003). These structural proteins are folded and entered in to the endoplasmic reticulum (ER) and transported to the ER-Golgi transitional slot (Masters, 2006). During the replication of coronavirus, substantial amounts of structural proteins are synthesized in order to assemble the progeny virions (Fung and Liu, 2014). They occupy the RNA genome which encodes structural proteins such as S protein, M protein, and N protein (Snijder et al., 2003).

**FIGURE 1:**
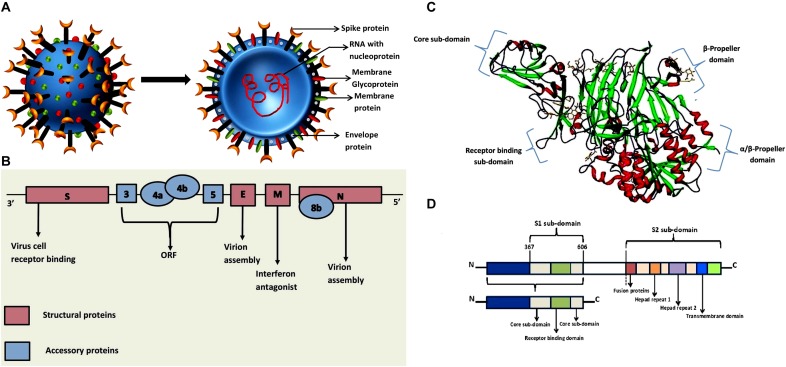
**(A)** Graphical representation of the structure of the MERS-CoV. The schematic representation shows that the structure of Middle East Respiratory Syndrome Coronavirus, which contains the RNA enclosed in the nucleocapsid within the envelope that is embedded with spike proteins, glycoproteins and membrane proteins (van den Brand et al., 2015). **(B)** Genome organization of MERS CoV. The genomic organization of MERS-CoV displays the viral genes S, 3, 4a, 4b, 5, E, M, 8b, and N. The genome encodes for five unique accessory proteins such as 3, 4a, 4b, 5 and 8b and four major structural proteins which are illustrated in the genome scheme (Gao et al., 2016). **(C)** Experimental structure of MERS-CoV complexes with human DPP4 (PDB ID: 4L72). DPP4 is an extracellular RBD domain, which comprises of both N- and C-terminal. The N-terminal is the β-propeller domain and C-terminal is the α/β hydrolase domain (Wang et al., 2013). **(D)** Genome organization of receptor binding domain (RBD) that illustrates the S1 and S2 sub-domains (Zhang et al., 2015).

The genome size of the first imported MERS-CoV strain was identified to be 30,114 nucleotide (nt) long, including the 3′and 5′ UTRs. This strain showed the genome structural ornaisation of typical betacoronavirus such as a 5′-untranslated region (UTR) (nt 1 to 272), replicase complex ORF1ab (nt 273 to 21508), S gene (nt 21450 to 25511), ORF3 (nt 25526 to 25837), ORF4a (nt 25846 to 26175), ORF4b (nt 26087 to 26827), ORF5 (nt26834 to 27508), E gene (nt 27584 to 27832), M gene (nt 27847 to 28506), N gene (nt 28560 to 29801), ORF8b gene (nt 28756 to 29094), and 3′UTR (nt 29094 to 30114). The two main poly-proteins such as *pp1ab* and *pp1a* are cleaved into 15/16 non-structural proteins (nsp) by 3C-like protease (3CLpro) and papain-like protease (PLpro). These proteases are cleaved from *pp1ab* along with other ORFs encoding nsp which are essential for activating RNA dependent RNA polymerase, helicase, exo-ribonuclease, endo-ribonuclease, and methyltransferase. These nsp were identified to be nsp12, nsp13, nsp14, nsp15, and nsp16. The nsp14 is necessary for proofreading the ubiquitous mutation of RNA virus (Ziebuhr et al., 2000; Snijder et al., 2003; Gorbalenya et al., 2006; Smith et al., 2013; Raj et al., 2014; Durai et al., 2015). Studies revealed that the accessory ORF proteins play an important role in MERS-CoV infection and pathogenesis (Menachery et al., 2017). This study showed that the absence of major accessory ORFs such as deletion of ORF3, -4a, -4b, and -5 (dORF3-5) played major role in the viral replication and pathogenesis. Further, the attenuation of the mutant dORF3-5 was found to be responsible for the dysregulated host responses such as augmented interferon (IFN) pathway activation, disrupted cell processes, and robust inflammation. Thus, the disruption of accessory ORFs probably provide platform to the attenuation of future emergent strains of MERS-CoV accessory ORF mutants. The accessory ORF functions can be targeted for both therapeutic and vaccine treatments in response to MERS-CoV and related group 2C corona viruses (Menachery et al., 2017). The graphical representation of the genome organization of MERS-CoV is illustrated in Figure 1. Coronaviruses have demonstrated the establishment of double membrane vesicles (DMVs) in the infected cells. Based on immune-histochemistry and electron microscopic studies, the coronavirus replicase proteins are co-localized with the DMVs and are assumed to be the site of replication/transcription complex (Fung and Liu, 2014).

An interesting fact of coronaviruses is that they display recombination, high rates of mutation, and propensity to cross species. One such event was reported during the 2002–2003 epidemic, where the transmission of SARS-CoV has occurred from Chinese horseshoe bats to human populations (Perlman and Netland, 2009). MERS-CoV has the capability to adjust new environments and acquire various virulence factors and enhance their ability to transfer these factors from human-to-human due to continues outbreak (Zumla et al., 2015). The phylogenetic analysis revealed that all human and camel MERS-CoVs were identified to be homologous to each other. Additionally, the hedgehog and bat MERS-CoVs demonstrated para-phyletic group to human and camel MERS-CoV clade (Zhang et al., 2016).

Kim (2016) reported that the occurrence of a mutant strain of MERS-CoV toward the human CD26 receptor during the South Korean outbreak. Furthermore, they isolated 13 new viral genomes in which, 12 possessed point mutation in the receptor-binding domain (RBD) of S protein (Kim, 2016). The S protein is suggested to be essential for the host tropism through its interaction with the host CD26 receptor (van Doremalen et al., 2014; Wang et al., 2014).

## Pathogenesis

### Viral Entry

The entry of MERS-CoV to the host is facilitated by its type-I transmembrane glycoprotein known as S protein (Xia et al., 2014). The RBD of MERS-CoV consists of S1 subunit which ranged from 367 to 606 amino acids and can be grouped into an external sub domain and core domain as shown in Figure 1C,D. The receptor-binding motif, V484 – L567 of RBD is situated at the external sub domain and the core sub domain consists of anti-parallel β-sheet (five-stranded) and six helices in the center. The two small β-strands make a globular fold. The core domain structure is balanced by three disulfide linkages. The MERS-CoV RBD external sub domain contains a β-sheet along with one small and three large strands which are organized in anti-parallel manner. The RBD core is attached to this region by special intervening loops and it binds to the core sub-domain by a clamp at the lower and upper regions. Most of the connecting loops and two small *3_10_* helices are located in the inner region of the sheet. The fourth disulfide linkage is stabilized among the residues starting from C503 to C526 and β6-strand linked with η3-helix. Three helices (HR1) at the center and three chains (HR2) near to the core promote the release of the viral particle into the cytoplasm and promote the progression of the infection (Durai et al., 2015).

**FIGURE 2:**
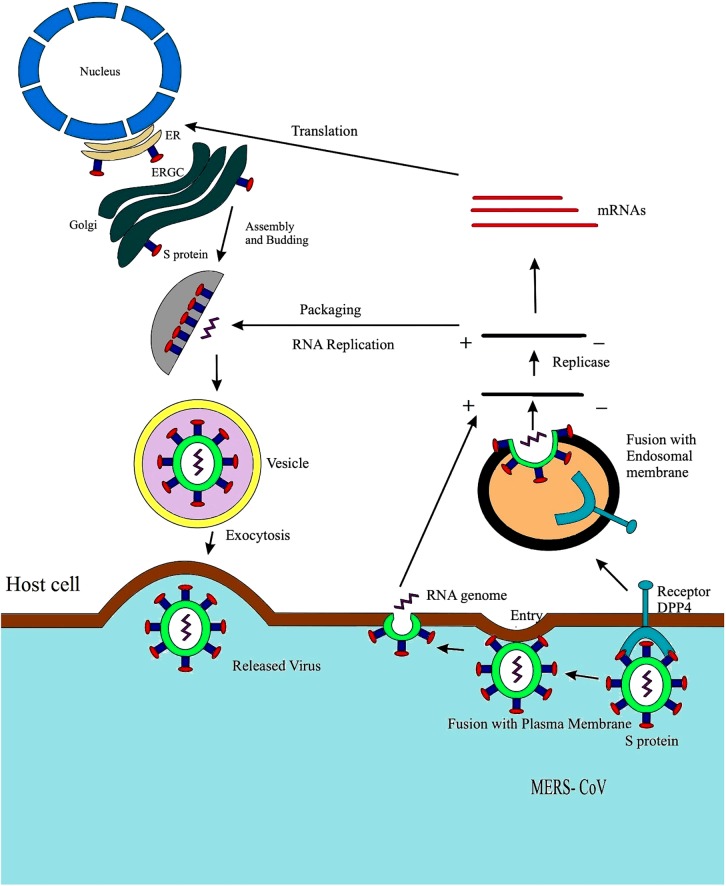
Life cycle of MERS-CoV displaying fusion with plasma membrane. The fusion of S protein to the plasma membrane of host cell, formation of a double membrane vesicle in the host cell, eventually releasing the RNA enclosed in the nucleocapsid followed by genome transcription. The viral RNA undergoes replication and transcription followed by the 4, 5, and 6 RNA synthesis and translation; the endoplasmic reticulum aids the assembly and packaging of virus particle forming a complete double membrane vesicle and lastly through exocytosis and MERS-CoV is released out of the host cell (Du et al., 2009).

Middle East Respiratory Syndrome Coronavirus can also enter the cell through an auxiliary pathway on the cell surface via transmembrane proteases. The binding of MERS-CoV into host cell and replication of the virus is shown in Figure 2. The host protease cleaves coronavirus S protein into two functional domains distinctive to each other at the N-terminal region (denoted as the S1 subunit) which comprised of RBD, and C-terminal portion (S2 subunit) comprised of a fusion peptide, two heptad repeats (HR2 and HR1) and the transmembrane (TM) domain (Weiss and Navas-Martin, 2005). The membrane fusion is conciliated by main conformational rearrangement which exposed the fusion peptide resulting in the development of six-helix bundle (6HB). The core of 6HB consists of a triple-stranded coiled like structure constructed by HR1s of the three spike subunits forming trimers; the HR2 elements are found to be packed within the grooves of the coiled coil in an anti-parallel direction. Owing to their central role in membrane fusion, it was reported that various antiviral peptides interfere the formation 6HB (Forni et al., 2015).

### Transmission and Clinical Presentation

The clinical presentation of MERS-CoV compasses from severe respiratory diseases to subclinical infections (Drosten et al., 2013; Hui et al., 2018). Infected patients often indicate the presence of hemoptysis, sore throat, fever, cough, shortness of breath, and other gastrointestinal symptoms such as diarrhea and vomiting (Albarrak et al., 2012; Assiri et al., 2013a,b; Guery et al., 2013; Health Protection Agency, 2013; Omrani et al., 2013). A low range of lower pulmonary infiltrate associated with viral pneumonia was observed in the radiograph of patients infected with MERS-CoV (Memish et al., 2013a; Wang, 2014). It was observed that greater than 60% of the initially reported cases of MERS-CoV infection, the patients experienced severe disease which demanded intensive care treatments like extracorporeal membrane oxygenation and mechanical ventilation. Hematological aberrations delineated for the clinical cases of neutrophilia (8%), lymphopenia (34%), thrombocytopenia (36%), and lymphocytosis (11%). While the conditions of kidney failure necessitate renal replacement therapy for a considerable number of MERS-CoV cases, several studies detected MERS-CoV antigens and particles in the renal tissues *in vivo*, which is the direct indication of the virus replication in renal tissues and long term acute infection (Yeung et al., 2016; Alsaad et al., 2018).

The first evidence of human-to-human transmission of MERS-CoV was reported in few cases in United Kingdom, when an adult male traveled to Saudi Arabia and transferred the infectious virus to two of his family members (Perlman and Netland, 2009). It was suggested that MERS-CoV infections were due to the introduction of the virus in humans and the close contact with camels was one of risk factors for the transmission of MERS as reported by World Health Organization [World Health Organization (WHO), 2016b].

**FIGURE 3:**
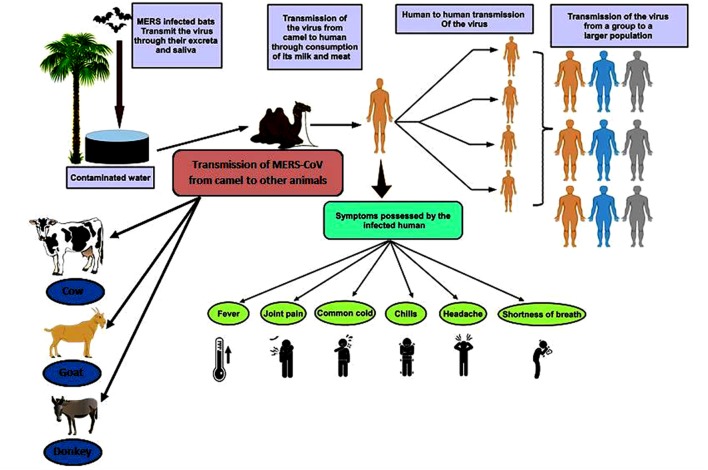
Transmission of MERS-CoV and symptoms possessed by infected human. The figure shows the path by which the virus is transmitted from the infected camel to the human and camel to various animals (Kandeil et al., 2019); further, this virus is transmitted to other human population via human to human transmissions (Shehata et al., 2016).

The transmission of MERS-CoV is determined as spasmodic, often healthcare associated, intra-familial, and required prolonged and close contact (Memish et al., 2013a,b, Memish et al., 2014a,b; Puzelli et al., 2013; Drosten et al., 2014). In a domestic study conducted with MERS-CoV infected patients, 14 out of 280 contacts of 26 positively indexed patients (antibody positive or RNA) suggested that the frequency of the transmission even during outbreaks was approximately 3% (Drosten et al., 2014). Several studies suggested that the virus was not self-sustained, as during local epidemic of MERS-CoV, which has not been readily transmitted to more than one human and has been reported in majority of human related cases (Bauch and Oraby, 2013; Breban et al., 2013; Cauchemez et al., 2014; Chowell and Nishiura, 2014). The mode of transmission of the virus is illustrated in Figure 3.

The serological analysis and extensive investigation of those potentially exposed to the patients suggested that there were no secondary infections [World Health Organization (WHO), 2013b]. As a minimum of 18 cases of asymptomatic MERS-CoV infections were reported in health care personals, the function of these subclinical cases in the transfer of infection was found to be uncertain (de Sousa et al., 2014).

### Molecular Mechanism of the Pathogenesis

While there were intensive studies on coronavirus research over the past 5 years, limited reports are available on the pathogenesis of MERS-CoV. Recent post-mortem histopathological studies revealed that the localization of viral particles in the pulmonary and extra pulmonary tissue of a 33-year-old male patient of T cell lymphoma, who acquired MERS-CoV infection (Ng et al., 2016; Alsaad et al., 2018). The histopathological analysis of the biopsies were collected from lung, brain, liver, heart, kidney, and skeletal muscle demonstrated that there were pulmonary diffuse alveolar damage, necrotizing pneumonia, acute kidney injury, portal and lobular hepatitis and myositis with muscle atrophic changes. Further, the viral particles were found to be localized in the pulmonary macrophages, pneumocytes, renal proximal tubular epithelial cells and macrophages infiltrating the skeletal muscles (Alsaad et al., 2018).

**FIGURE 4:**
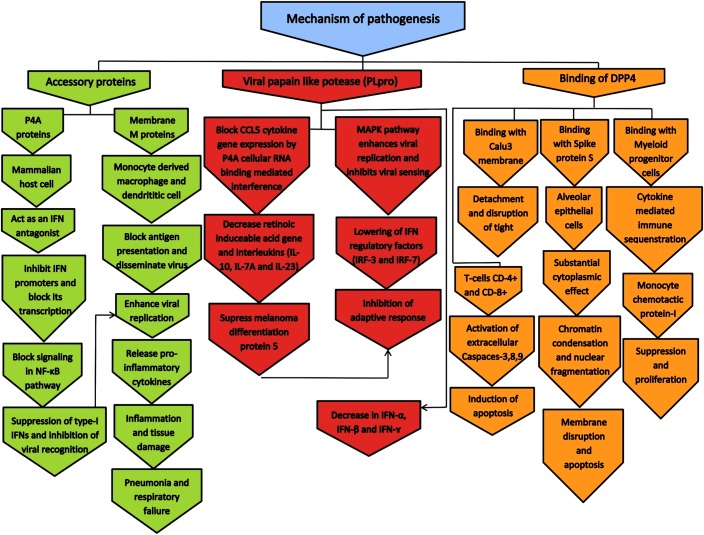
The molecular mechanism of pathogenesis by MERS-CoV in human and animals. The pathogenesis mechanism is mainly occurred by DPP4, papian like protease PLpro, and accessory proteins such as p4a and membrane M protein (Durai et al., 2015).

Studies suggested that the pathogenesis of MERS-CoV in human and animals are mainly due to three mechanisms such as DPP4 (dipeptidyl peptidase-4) mediated mechanism, papain like protease PLpro mediated mechanism and accessory proteins like p4a and membrane M protein mediated mechanism. Hence, these proteins are probably considered as one of the potential therapeutic targets for MERS-CoV infections (Durai et al., 2015). The molecular mechanisms exhibited by various modes of pathogenesis are illustrated in Figure 4.

## DPP4 Mediated Mechanism

DPP4 is a type-II transmembrane glycoprotein consists of approximately 766 amino acids and function as major receptor for MERS-CoV. The three dimensional structural analysis revealed that this receptor has α/β-hydrolase domain and a β-propeller domain with eight blades that helped in the binding of receptor binding domain in MERS-CoV (Raj et al., 2014). The crystal structure of DPP4 is illustrated in Figure 1C. A DPP4 binding protein, adenosine deaminase, is considered as one of the major inhibitors of the S protein of MERS-CoV (Drosten, 2013).

A number of cell lines such as human-derived HFL, Calu-3, Caco-2, Huh-7, HEK, His-1 cell lines, CD8+, and CD4+ that are susceptible to MERS-CoV were reported (Chan et al., 2013b; Shirato et al., 2013; Chu et al., 2016). Two S protein ecto-domains can be divided into fusion-catalyzing domains (FD) and RBD. The S RBDs bind to DPP4 after which the FDs are exposed through unfolding. Further, the cellular and viral membranes were joined together by refolding (Raj et al., 2013). The unfolding FD required the cleavage of S protein by host proteases. Out of various proteolytic cleavage sites between the FD and RBD, the cleavage at S1/S2 site is essential for the infection of Calu3 cells by MERS-CoV (Park et al., 2016).

It has been revealed that upon infection, monocyte chemo attractant protein-1 and IFN-γ-inducible protein-10 were induced and suppressed the proliferation of human myeloid progenitor cells (Zhou et al., 2014). MERS-CoV can infect T-cells from human lymphoid organs and causes the peripheral blood inducing apoptosis by intrinsic and extrinsic pathways (Chu et al., 2016). A study conducted by Al-Qahtani et al. (2017) suggested that the inhibition of DPP4 mitigated the induction of PPARγ (the transcriptional repressor) and IRAK-M (negative regulator of TLR signaling) which indicated that the immunosuppressive action of S glycoprotein regulated by DPP4.

## Papain Like Protease PLpro Mediated Mechanism

Upon entry into the cells, two ORFs at the 5′-end of the viral genome are translated into two poly-proteins. They are processed by two viral proteases, *PLpro* and 3C-like proteinase (3CLpro) and form 16 nonstructural proteins which are necessary for the membrane-associated replication complex. *PLpro* is located in nonstructural protein 3. *PLpro* was found to be multifunctional enzymes with deISGylating (removal of ISG15 conjugates from host cell factors) and de-ubiquitinating (cleavage of ubiquitin from host cell factors) properties in addition to protease activity in many coronaviruses (Clasman et al., 2017). This resulted in the host antiviral immune response being antagonized and promoting the viral replication. *PLpro* can inhibit the mitochondrial anti-viral signaling protein induced IFN-β reporter activity and reduce TNF-α induced NF-κB reporter activity. Hence, MERS-CoV *PLpro* is considered to be important antiviral target (Mielech et al., 2014; Harrigan et al., 2018).

The coronavirus genome is encoded by two ubiquitin-like domains (Ubl1 and Ubl2), out of which Ubl2 is considered to be an important component which influences the *PLpro* activity. Clasman et al. (2017) reported that *PLpro* has not showed the deISGylating, de-ubiquitinating or protease activities in the absence of Ubl2 domain. There were no variations in the process of inhibition, substrate specificity, catalytic efficiency and thermal stability (Clasman et al., 2017).

## Accessory Proteins Mediated Mechanism

The viral genome is recognized by melanoma differentiation-associated protein-5 (MDA5), retinoic acid inducible gene-1 (RIG-1) and endosomal toll-like receptor 3 (TLR3) as pathogen-associated molecular patterns. This recognition resulted in the formation of type-1 interferon (IFN1). As an evasion mechanism, virus synthesize proteins that hinder the production IFN1 in the pathway (Zinzula and Tramontano, 2013).

In the absence of MDA5 and RIG-1, MERS-CoV used the protein such as 4a (p4a) which contain RNA-binding motif for binding (Rabouw et al., 2016). By a direct binding, this motif masks the dsRNA of MERS-CoV. The p4a possesses αβββα fold with the β1-β2 loop and α1 helix which bind to the minor groove of dsRNA. The mutational studies revealed that p4a-dsRNA complex stabilized by the amino acids such as K27, W45, K63, and K67 (Batool et al., 2017). In a study carried out by Canton et al. (2018) suggested that, NF-κB remained in the cytoplasm of MERS-CoV infected cells while 4b was found to be attached with the nucleus. In the absence of 4b, expression of pro-inflammatory cytokines was observed as NF-κB translocated to the nucleus. This was also observed in the case of 4b mutants that devoid of nuclear localization signal, indicating that nuclear localization signal mediated process of 4b is essential for NF-κB expression (Canton et al., 2018).

As a component of the viral envelope, M protein is involved in various functions such as viral assembly along with other proteins such as E, S, N. The M, E, and S proteins interact with N protein in the ER-Golgi complex. This interaction hinders the fusion of viral and cellular membranes (Mustafa et al., 2018). It was observed that the expression of M protein in MERS-CoV might suppressed the type-I IFN expression in response to poly (I: C) induction or Sendai virus infection. This reaction was detected to be specific for the activation of IFN regulatory factor 3 (IRF3), however, not activated the nuclear factor-κB. The interaction of MERS-CoV M protein with TRAF3 and the association of disrupted TRAF3–TBK1 lead to reduced activation of IRF3 (Lui et al., 2016).

## Host Immune Response

Middle East Respiratory Syndrome Coronavirus can readily infect human respiratory epithelial cells, macrophages, T-cells, dendritic cells and can influence the production and induction of pro-inflammatory cytokines and chemokines (Zhou et al., 2015). Studies on *ex vivo* human lung tissue and respiratory epithelial cell lines showed that the induction of antiviral interferons followed by MERS-CoV infection by stimulated epithelial cells was found to be limited. A study by Menachery et al. (2014) described that the cells infected with MERS-CoV demonstrated distinctly altered chromatin structures such as repressive histone markers, that could limit the transcription factors from binding to the promoter regions of interferon-stimulated gene. The changes in DNA methylation found to be one of the reasons for down regulation of IFN-γ–associated antigen-presenting gene after the infection (Menachery et al., 2018). MERS-CoV proteins such as 4a, 4b, M, and PLpro were found to be suppressed the induction of interferons (Yang et al., 2013; Mielech et al., 2014; Canton et al., 2018). Further, the induction of pro-inflammatory cytokine response in these cells was found to be limited. A delayed induction of pro-inflammatory cytokines/chemokines such as IL-6, IL-8, and IL-1β were observed upon the viral infection (Lau et al., 2013; Canton et al., 2018).

Middle East Respiratory Syndrome Coronavirus could infect and replicate in human monocyte-derived macrophages and immature monocyte-derived dendritic cells, while it failed to perform similar functions in mature monocyte-derived dendritic cells. This indicate that the maturation restrict the viral replication. Immature dendritic cells were unable to stimulate T-cell even after the antigen uptake, delaying the activation of T-cells and permitting further viral replication (Cong et al., 2018). The infection of human macrophages induced the expression of pro-inflammatory cytokines/chemokines such as MCP-1/CCL-2, IFN-α2, IFN-γ, MIP-1α/CCL-3, IP-10/CXCL-10, RANTES/CCL-5, IL-8, TNF-α, IL-12p40, and IL-6 (Zhou et al., 2015; Cong et al., 2018). The infected dendritic cells induced the expression of IL-12, RANTES/CCL-5, IP-10/CXCL-10 and IFN-γ, however, showed low expression of IFN-α and no expression of IFN-β (Zhou et al., 2015).

T-cells can be directed to the infection site by type-I IFN stimulated secretion of IL-10 and CXCL10. Due to their uncontrolled and strong expression, the expression of IFN-γ and IL-12 were inhibited and restricted the activation of T-helper cells. With the added effect of down-regulated antigen-presentation pathways, the activation of T cells is inhibited. Hence, the sequestered T cells failed to target the virus (Ying et al., 2016). Previous studies revealed that when the serum samples and bronchoalveolar lavage were analyzed from two patients, it was observed that the patients with low level secretion of IFN-α were failed to survive and the patients displayed high level secretion of type-I IFN were survived (Faure et al., 2014).

## Animal Models

The animal models are one of the vital components for translation of the findings from drug discovery process from bench to bedside. These animal models are indispensable for the deeper understanding of any diseases and safe and protective vaccine development process (Lorenzen et al., 2015). The main criteria for the validation of animal models in preclinical studies are face validity, predictive validity and target validity. The face validity means that the proper similarity between the animal model and the human disease in terms of the biology and symptoms of the disease. However, assessing face validity is a tedious task due to the lack of understanding on the core biology of the disease symptoms. Predictive validity means the display of clinical interventions which exhibited similar effect in the animal models. This is also very difficult to achieve due to the incomplete correlation between animal models, the mechanism of human diseases and the incapability of standard drugs to active in many animal models. Target validity is another essential component for the validation of animal models in which the target under study must have an analogous function in the disease model as in the clinical condition (Denayer et al., 2014).

An ideal animal model is the one where an immunocompetent animal, on receiving the challenge virus at a realistic dose through a suitable inoculation route, is able to replicate the characteristics of a human disease as closely as possible (Doremalen and Munster, 2015). Ferrets, hamsters, mice and other small laboratory animals are not ideal animal models for MERS-CoV owing to the variations in DPP4 receptor. The rhesus macaque and the common marmoset are the first non-human primate species (NPS) to be used as the animal models for MERS-CoV. Glycosylation of mouse DPP4 (mDPP4) prevents its binding to S protein of MERS-CoV. Hence, transgenic hDPP4 (human DPP4) mice were developed as animal model (Peck et al., 2015). The major advantages and limitations of MERS-CoV animal models are listed in Table 2.

**Table 2:** Advantages and limitations of MERS-CoV experimental animal models.

Species	Advantages	Limitations	Reference
Rabbit	Readily available and easy to handle	Animal-to-animal transmission studies are not available No clinical disease, Low viral titers in tissues upon infection Develop mild pulmonary lesions	Haagmans et al., 2015; Vergara-Alert et al., 2017
Rhesus macaques	Human-specific reagents available for immunologic analysis Immune and respiratory systems similar to humans; clinical disease similar to humans Useful for confirming vaccine efficacy testing	Limited availability and expensive Expert husbandry requirements Animal-to-animal transmission studies are not available Transient disease Ethical concerns	Baseler et al., 2016; Yu et al., 2017
Common marmoset	Model severe, potentially fatal MERS-CoV infection Some human-specific immunological reagents cross-react Respiratory and immune systems similar to humans; clinical disease similar to humans Useful for confirming antiviral and vaccine efficacy testing	Limited availability and expensive Expert husbandry requirements Ethical concerns Animal-to-animal transmission studies are not available	Vergara-Alert et al., 2017; Yu et al., 2017
hDPP4-transgenic mice	Model severe, potentially fatal MERS-CoV infection Easy to handle Reagents available Useful for screening antivirals and vaccines	Global over expression of hDPP4	Baseler et al., 2016; Coleman et al., 2017


### Mice

Studies suggested that the glycosylation of certain amino acids prevented the binding of mouse DPP4 (mDPP4) to S protein of MERS-CoV (Doremalen and Munster, 2015). The first transgenic MERS-CoV mouse model was established by Agrawal et al. (2015). The mice expressed hDPP4 and developed severe respiratory MERS-CoV infections. The transgenic mice infected with an intra-nasal dose of 10^6^ TCID50 of MERS-CoV developed pneumonia, weight loss, acute pulmonary viral infection, ruffled fur, squinting, and death within few days (Agrawal et al., 2015). A transgenic mouse model developed by Zhao et al. (2015) showed that limited utility for pathogenic studies due to the systemic dissemination and development of severe neurological diseases. The other lung infection models were lack mortality and weight loss, however, developed lung pathology in response to 50% TCID^50^ of MERS-CoV (Tao et al., 2015).

Recent studies suggested that the transgenic mice models were developed where the full-length mDPP4 gene replaced by hDPP4 (Coleman et al., 2017). The infection and replication of MERS-CoV was observed in the lungs, however, disseminated slow impact to the other organs during the post injection with dose range of 102–105 PFU. The pathology of infected mice showed pneumonia-like symptoms (Coleman et al., 2017).

The CRISPR-Cas9 gene editing tools were used to produce mice susceptible to MERS-CoV infection by modifying their genome sequence (positions 288 and 330) homologous to the sequence of hDPP4. In certain mice model the characteristic symptoms such as pulmonary hemorrhage, low survival, decreased pulmonary function and weight loss were observed. The 288-330+/+MERS-CoV mouse model demonstrated severe respiratory infection as observed in humans (Cockrell et al., 2016).

### Rhesus Macaques and Marmosets

Upon infection with MERS-CoV, rhesus macaques and marmosets were developed moderate respiratory disease which are comparable with mild human MERS-CoV infection. As observed in human cases, they also showed complete blood count abnormalities. Rhesus macaque and marmoset models were found to be suitable for pathogenic studies of mild infections. Both the models can be used for the efficacy testing of prophylactic and therapeutic countermeasures (Baseler et al., 2016).

Yu et al. (2017) reported that when 2-3 old rhesus macaques and common marmosets were intra-tracheally injected with hCoV-EMC, hematoxylin and eosin stained tissues of the infected model demonstrated lesions primarily in the lungs with various degrees of inflammation, hemorrhaging, pulmonary oedema, interstitial pneumonia, eosinophil infiltration and necrosis in bronchial epithelial cells and pneumocytes. The lungs of the infected marmosets demonstrated widespread pulmonary oedema and hemorrhaging. The oedematous alveolar cavities showed fibrinous exudates and neutrophil infiltration. In both the cases, DPP4 was widely expressed in alveolar macrophages and type-I and II pneumocytes were also actively involved. The pathological changes as a result of viral infection were not observed in any other organs of the infected animal (Yu et al., 2017).

### Rabbits

In most cases, MERS-CoV causes severe infection in the lower respiratory tract in humans. Nasal swabs collected from the infected rabbits demonstrated the presence of MERS-CoV. They generally develop respiratory diseases such as asymptomatic and mild human infections as sometimes observed in immune-competent patients (Haagmans et al., 2015).

Recently, Houser et al. (2017) revealed that the infected New Zealand rabbits were developed asymptomatic pulmonary infections with high levels of viral antigens and RNA. Multiple lung lobes showed peri-vascular inflammation. The antibodies developed by the rabbits lacked neutralizing activity, and as a result the animals were susceptible to re-infection. In fact, enhanced pulmonary inflammation was observed after re-infection. However, re-infection elicited the neutralizing antibodies (Nabs). Hence, it was suggested that upon re-infection, there might be greater risk of severe lung disease in those who do not develop neutralizing antibody response, or those whose neutralizing antibody titers have decreased after the initial infection (Houser et al., 2017). The clinical signs and lesions along with the occurrence of MERS-CoV RNA and antigens observed in MERS-CoV infected animal models are shown in Table 3.

**Table 3:** Clinical signs and lesions along with the occurrence of MERS-CoV RNA and antigens as seen in MERS-CoV infected animal models.

Animal model	Clinical signs	Lesions	Occurrence of MERS-Cov RNA	Occurrence of MERS-antigen	Reference
Rabbit	Asymptotic	Rhinitis with necrosis	Lung, upper respiratory tract, lymph nodes	Type I and II pneumocytes	Haagmans et al., 2015
Rhesus Macaques	Mild to moderate respiratory disease	Interstitial pneumonia	Lung, lymph nodes, upper respiratory tract	Type I and II pneumocytes, alveolar macrophages	de Wit et al., 2013a,b; Yao et al., 2014; Yu et al., 2017
Common Marmoset	Mild to severe respiratory disease	Broncho-interstitial pneumonia	Lung, blood, lymph nodes, visceral organs, upper respiratory tract	Type I pneumocytes, alveolar macrophages	Falzarano et al., 2014; Yu et al., 2017
hDPP4- transgenic mice	Severe fatal respiratory disease	Broncho-interstitial pneumonia	Lung, brain, visceralorgans	Type I and type II pneumocytes	Agrawal et al., 2015


## Diagnostic Approaches

The primary diagnosis of MERS-CoV infection was performed by molecular techniques such as real-time reverse transcriptase PCR (RT-PCR) (Corman et al., 2012a), reverses transcription–loop-mediated isothermal amplification (RT-RTPA) (Shirato et al., 2014) and reverses transcription-recombinase polymerase amplification (RT-LAMP) (Abd et al., 2013). Numerous serological assays were used to detect MERS-CoV or closely related viruses in seropositive camels; these tests were protein microarrays like indirect enzyme-linked immunosorbent assay (ELISA), recombinant spike immunofluorescent assay, spike pseudoparticle neutralization and microneutralization assay (Buchholz et al., 2013; Perera et al., 2013). However, none of the serological assays showed evidence of the precise occurrence of MERS-CoV in camels (Song et al., 2015). Recent study reported that a rapid and specific assay for the detection of MERS-CoV such as nucleic acid visualization technique which combine the reverse transcription loop-mediated isothermal amplification technique and a vertical flow visualization strip (RT-LAMP-VF) to detect the N gene of MERS-CoV (Huang et al., 2018). The study suggested that in comparison with the real-time RT-PCR (rRT-PCR) method recommended by the World Health Organization, the RT-LAMP-VF assay is easy to perform and the rapid detection of MERS-CoV within 35 min was achieve by this approach (Huang et al., 2018).

An assay was developed based on the detection of N protein of MERS-CoV (Song et al., 2015). The NP protein preferred as better target than the S protein. In this type of assay, firstly, the antigen binds to gold labeled monoclonal antibodies which lead to the formation of antigen-antibody complex. The complex further passed through nitrocellulose membrane and bind to monoclonal antibody at the test region and formed band which could be conveniently analyzed (Tang, 2014).

Currently, multiple purified monoclonal antibodies are available in clinical and preclinical studies for the development of antimicrobials (Rockx et al., 2010). Studies suggested that two human antibodies (REGN3048 and REGN3051) are known to bind with MERS-CoV RBD and prevent the interaction of cellular receptor DPP4 with S protein and effectively neutralize MERS-CoV infection (Pascal et al., 2015). The antibodies such as REGN3048 and REGN3051 showed no binding with S protein, thus, employed as effective combination for inhibiting MERS-CoV (Aderem et al., 2011). REGN3048 and REGN3051 co-interact with MERS-CoV RBD, which suggested that these antibodies bind to discrete epitopes as they bind to regions of MERS-CoV S protein that are conserved during the natural evolution of the virus. These two antibodies were blocked entry of the virus into susceptible cell lines and neutralize the infection (Pascal et al., 2015).

The diagnostically relevant variations in the neutralization activity have not been detected in several isolates of MERS-CoV. In order to detect the serological response to a specific type of single MERS-CoV serotype, specific protein based sero-assays are essential to be performed (Muth et al., 2015). The development of potential serological assays demand well delineated human or animal sera and antibodies specific to MERS-CoV (Meyer et al., 2014).

## Vaccines and Therapeutics

Previous studies revealed that both cellular and humoral responses are required for the complete protection against MERS-CoV infections (Faure et al., 2014; Park et al., 2015; Corman et al., 2016). Nabs and T-cell responses are the main agents in providing protective immunity against coronaviruses. In a study by Zhao et al. (2014) reported that MERS-CoV cleared by mice which lack B-cells while those deficient in T-cells failed to perform similar functions. Further, since virus-specific humoral responses were decreased over time, developing a vaccine which could induce long-lived memory T-cell response considered to be beneficial. However, any vaccine compounds which are able to induce both T and B-cell responses are required for immune protection (Okba et al., 2017).

### Current Vaccine and Antiviral Candidates

MERS-CoV vaccine candidates are still under development with DNA based, protein based, live attenuated and vectored-virus based vaccines (Arabi et al., 2017). Out of the four structural proteins and sixteen non structural proteins encoded by the viral genome, S protein showed the highest immunogenicity which induced T-cell responses. In most of the MERS-CoV, Nabs target the RBD of the S protein. The variations in amino acid sequences of the S protein detected amongst MERS-CoV is limited, thus, designing a vaccine against one strain most likely to be protected against all the other circulating strains (Okba et al., 2017). The S1 protein located outside the RBD probably considered as potential drug target to develop therapeutic strategies against MERS-CoV. RBD-specific mAbs are considered to be displayed greater neutralizing activity than those were targeted the S1 or S2 region outside RBD (Du et al., 2013).

DPP4-targeting therapeutic agents are capable of blocking the interaction between MERS-CoV RBD and DPP4, and therefore inhibit MERS-CoV infection. The anti-DPP4 (CD26) antibodies such as 1F7, 2F9, and YS110 prevent the entry of MERS-CoV into susceptible cells, obstructing the virus infection (Ohnuma et al., 2013). Additionally, DPP4-binding protein adenosine deaminase (ADA) competes the binding with MERS-CoV RBD to DPP4, specifically at crucial residues such as Q286 and L294, defining their role as a naturally occurring antagonist (Raj et al., 2014).

Recent studies revealed that the DNA vaccines are the safest vaccines for immune-compromised patients. They possessed comparatively greater amounts of DNA (e.g., 0.5–2 mg) thus requires multiple inoculations to induce potent immune responses in rhesus macaques and mice (Wirblich et al., 2017). Chi et al. (2017) developed a DNA vaccine which encoded the first 725 amino acids of S protein in MERS-CoV which induced the cell mediated and antigen-specific humoral immune responses in mice. Post three immunizations, it was observed that high titers of Nabs (up to 1: 104) were produced without any adjuvant. A prophylactic DNA-plasmid vaccine known as GLS-5300, which encodes for S protein, is the first MERS-CoV vaccine to be considered for the human trials developed by GeneOne Life Inc., which has been enlisted in the on-going list of vaccines by WHO [Rabaan et al., 2017; World Health Organization (WHO), 2018b].

The vaccines that imitate the natural infection, such as recombinant viral vectors or live-attenuated virus present the antigens in natural ways which further stimulate the activation of humoral and cell-mediated immunity to the native protein conformation. As the live attenuated viruses have been the most immunogenic platforms available, they exhibited the capability to present numerous antigens across the viral life cycle in their native conformations (Henderson and Moss, 1999). Agrawal et al. (2016) vaccinated the mice with an inactivated MERS-CoV vaccine and which demonstrated hypersensitivity risk factors in MERS-CoV infection. Although there were extensive research in the last few years, limited anti-MERS-CoV therapeutics have been approved for human use. Originally, conventional IFAs were employed in the human sero-surveys and these relied on the MERS-CoV infected cell cultures. These cell cultures were the primary antigen sources for detecting the presence of human anti-MERS-CoV Nabs, IgM, and IgG in human samples (Corman et al., 2012b). Further, it was reported that human monoclonal antibody m336 demonstrated significant neutralizing properties against MERS-CoV *in vitro* conditions (Houser et al., 2016).

Recent studies revealed that there were few antiviral agents in the developmental stage. It was reported that the antiviral drug chlorpromazine demonstrated strong anti-MERS-CoV potential; however, the high cytotoxicity of the drug limiting the potential window for drug utilization. Similarly, another drug Toremifene showed moderate activity when tested in antigen presenting cells, however, the high cytotoxicity of this drug also narrowing its application as a therapeutic remedy against MERS-CoV (Cong et al., 2018). Further, studies suggested that a nucleoside analog GS-5734 known as Remdesivir, which showed potential antiviral activity against human and zoonotic CoVs *in vitro* and the drug inhibited MERS-CoV at 50% effective concentration values (EC50) (Agostini et al., 2018).

## Novel Therapeutic Approaches

The conserved nature of the N protein was used to develop vaccines that can induce an adaptive immune response against these proteins. In a study by Zhao et al. (2016) reported that a vaccine candidate induced airway memory CD4+ T cell response against N-specific epitopes of MERS-CoV. In another study suggested that vaccination with recombinant N-terminal domain (rNTD) triggered greater T-cell response than the mice vaccinated with rRBD. The neutralizing activity was also observed in the sera (Jiaming et al., 2017). Recent study revealed that the combination of fusion inhibitory peptide which targets the protein HR1 domain of MERS-CoV S2 and a neutralizing antibody specific for the S1 protein RBD showed synergistic therapeutic activities against MERS-CoV (Wang et al., 2019). Further, the researchers have developed novel neutralizing nanobodies (Nb) which specifically bind to the RBD of MERS-CoV S protein. The Nb was found to be interacted with the conserved domain of MERS-CoV RBD with high affinity and blocked binding of RBD to the receptor of MERS-CoV (Zhao et al., 2018).

Some immunodominant epitopes within the protein structure may contribute negatively to the neutralization activity of the vaccine. For example, a short region of RBD was able to induce higher titer of IgG when compared to other longer regions (Ma et al., 2014). The negative effects of these epitopes can be surmounted by immunofocusing; mapping of the most neutralizing RBD fragment and eliminating unnecessary fragments. This can help in focusing the immune response to important epitopes (Ma et al., 2014). Pallesen et al. (2017) suggested that the structure-based design of S proteins improved their immunogenicity where the high titers of Nabs were observed against MERS-CoV. The major categories of vaccines developed and tested against various animal models are shown in Table 4.

**Table 4:** Categories of vaccine developed against MERS-CoV, their potential candidate, and their target long with their immunological response and efficacy.

Vaccine categories	Vaccine candidate	Target antigen	Animal model	Immunological response	Efficacy	Reference
Protein based	S1	S1	Mice	Nab	Not tested	Wang et al., 2015
	rRBD-Fc	S377-588	Ad/hDPP4-mice	T-cell, Nab	Protective	Ma et al., 2014
	rNTD	S18-353	Ad/hDPP4-mice	T-cell, Nab	Protective	Jiaming et al., 2017
Recombinant viral vectors	BNSP333-S1	S1	Ad/hDPP4-mice	Nab	Protective	Wirblich et al., 2017
	S377-588-Fc	S	Ad/hDPP4-mice	T-cell, Nab	Not tested	Tang et al., 2015
	VRP-S	S	288/330+/+mice	Nab	Protective	Cockrell et al., 2016
DNA vaccine	pcDNA3.1-S1	S1	Ad5/hDPP4-mice	T-cell, Nab	Protective	Chi et al., 2017
	pVax1-S	S	Non human primates	T-cell, Nab	Protective	Muthumani et al., 2015


### Therapeutic Screening by Computational Biology

The technological progression is reflected upon a series of genome wide molecular screening platforms and computational biology approaches that provided novel insights to prompt response against emerging viral diseases. The prototype of these approaches included the *in vivo* animal model, tissue culture model, human challenge model, and vaccine studies. These model systems are anxious by challenges, preferably yielding a spectra of the severity of the diseases like lethal versus sub-lethal in order to increase divergence for downstream data mining and modeling (McDermott et al., 2011; Drosten et al., 2013; Law et al., 2013). A diversity of computational methodologies and network approach were used to detect the regulatory networks of various viral systems by *de novo* studies (Hou and Zhao, 2013; Mitchell et al., 2013).

Recently, studies have shown that peptides can be utilized as highly potent signal transduction agents for various viral infections. It has been suggested that antimicrobial peptides (AMPs) can be used as potential therapeutic option against MERS-CoV infections (Mustafa et al., 2018). Computational biology approach in combination with virology can be employed to design novel peptide based therapeutics against various strains of MERS-CoV with high efficacy. Recent studies have suggested that repurposing of existing and clinically approved anti-viral peptides can be used as promising lead molecules for the development of novel anti-MERS-CoV agents (Mustafa et al., 2018).

The RBD and S protein based vector vaccines are considered to be one of the efficient targets for designing MERS-CoV vaccine, as it comprised of multiple neutralizing epitopes which offer higher potential to prompt Nabs against the virus (Shi et al., 2015). The vast developments in computational biology have led to the new line of immunoinformatics databases and tools, which are other operative ways for vaccine development (Terry et al., 2015). The screening of dominant immunogens based on the availability of genome sequence data is crucial for designing novel vaccines, which is assisted by immunoinformatics (Firbas et al., 2006). The epitope directed lead design was one of the critical steps involved in the lead identification by immunoinformatics approaches (Shi et al., 2015). MHC-I restrained CD8+ cytotoxic T-lymphocytes (CTLs) identification provides profound insight in designing novel antiviral agents (Terry et al., 2015).

In conclusion, MERS-CoV infection continued to be fatal disease and has emerged as one of the epidemic concern. Irrespective of the various studies conducted to determine the distribution, the exact intermediates remain unidentified. There are various animal models available which provided profound insight in understanding the transmission and pathogenecity of the virus in human. The common pathogenesis mechanisms of the virus such as DPP4, PLpro, accessory proteins like p4a and membrane M protein provided significant insight for the screening of novel drug targets for vaccine development. The detailed understanding of the binding mechanism of various inhibitors toward the structural, non-structural, and accessory proteins of the virus probably provide profound insight for lead development. The integration of genome analysis, proteomics studies, immunoinformatics, and systems biology approaches on various animal models have made the recognition of new targets and lead molecules much easier in than the approaches available during earlier time. By considering MERS-CoV infections as one of the great public threats, there is high demand for undertaking the coronavirus research at the deeper molecular level to understand the mechanism of viral infection, development of advanced and rapid detection methods and futuristic therapeutic strategies to combat MERS-CoV infections.

## Author Contributions

SS, SC, SP, SK, and VS collected data and prepared the complete manuscript. Further, SS reviewed, revised, and edited the manuscript.

## Conflict of Interest Statement

The authors declare that the research was conducted in the absence of any commercial or financial relationships that could be construed as a potential conflict of interest.
